# Editorial: Nutraceuticals: transforming conventional foods into dietary medicine for the clinical management of neurodegenerative diseases and cancers

**DOI:** 10.3389/fnut.2024.1349492

**Published:** 2024-01-30

**Authors:** Syed Amir Ashraf, Mitesh Patel, Mohd Adnan

**Affiliations:** ^1^Department of Clinical Nutrition, College of Applied Medical Sciences, University of Ha'il, Ha'il, Saudi Arabia; ^2^Research and Development Cell, Department of Biotechnology, Parul Institute of Applied Sciences, Parul University, Vadodara, India; ^3^Department of Biology, College of Science, University of Ha'il, Ha'il, Saudi Arabia

**Keywords:** nutraceuticals, functional food, probiotics, cancer, neuro-inflammation, neurodegenerative disorder

Over the last few years, there has been a growing understanding and knowledge concerning human health and its association with foods. Additionally, the role of natural compounds, such as bioactive compounds with their possible function as health promoters has been found to be very important. As a result, diet-based strategies for the management and prevention of various chronic disorders including neurodegenerative diseases and cancer have become more meaningful for the development of nutraceuticals. Therefore, considering the fact this Research Topic of Frontiers in Nutrition aims to cover the concept of nutraceuticals and their significance in human health, which could serve as an alternative therapy or nutritional pharmacotherapy for the management of several clinical complications such as neurodegenerative disorders and cancer. Under this Research Topic, four articles (one original research, one review, one mini review, and one systemic review), covering the current updates for the development of nutraceuticals for targeted therapy in cancer and neurodegenerative diseases were published.

First paper addresses the strategic formulation of human probiotics, and allows the reader to walk along the journey that metamorphoses commensal microbiota into target-based probiotics. They recapitulate what are probiotics, their history, and the main mechanisms through which probiotics exert beneficial effects on the host. Here authors revisit the notion of host-adapted strains carrying niche-specific genetic modifications. Lastly, this paper emphasizes the strategic development of target-based probiotics using host-adapted microbial isolates with known molecular effectors that would serve as better candidates for bio-prophylactic and bio-therapeutic interventions in disease-susceptible individuals (Idrees et al.).

Second article explore that, does probiotic mitigate age-related neuro-inflammation leading to improved cognitive outcomes? As there are various changes takes place in the brain structure and cognitive function, and these are considered as a natural part of aging. However, in some cases these changes are more severe resulting in mild cognitive impairment (MCI) or Alzheimer's disease (AD). Here, author evaluated and concluded that there are preliminary evidences, which suggests that specific probiotics may improve cognitive function, particularly in those with MCI, but not very convincing. A larger, multi-location studies design specific to assess the effect of probiotics on cognition alongside the analysis of biomarkers of systemic inflammation, gut permeability and microbiota composition are needed (Anderson).

Third paper investigated the anti-cancer potential of synergistic phytochemical, and its combinations are influenced by the genetic profile of prostate cancer cell lines. They determined the effect of seven dietary phytochemicals such as quercetin, curcumin, genistein, indole-3-carbinol (I3C), equol, resveratrol and epigallocatechin gallate (EGCG), alone and in all paired combinations on cell viability of the androgen-responsive, pTEN-null (LNCaP), androgen-independent, pTEN-null (PC-3) or androgen-independent, pTEN-positive (DU145) prostate cancer (PCa) cell lines. Furthermore, the study revealed the synergistic potential with all the tested combinations. In LNCaP and PC-3 cells, I3C mediated maximum synergy with five phytochemicals, while genistein was maximally synergistic with EGCG. In contrast, DU145 cells showed resveratrol mediated maximum synergy with equol, EGCG and genistein, with I3C mediating maximum synergy with only quercetin and curcumin. Knockdown of pTEN expression in DU145 cells abrogated the synergistic effect of resveratrol without affecting the synergy profile of I3C and quercetin. They conclude that patterns of synergistic affect were dependent on tumor cell genotype, and are independent of androgen signaling, but are dependent on pTEN (Gano et al.).

Fourth and final paper presents a systematic review to explore the consumption of fish and risk of prostate cancer or its mortality. They also explore a dose–response meta-analysis of prospective cohort studies. Authors included the prospective cohort studies that examined the associations of fish intake with the risk of prostate cancer, its mortality, and cancer progression. In total, 25 prospective cohort studies, recruiting 1,216,474 men, were included in the systematic review, and 22 studies were included in the meta-analysis. During the follow-up periods, ranging from 6 to 33 years, a total of 44,722 cases of prostate cancer were recorded. The comparison between the highest and lowest intakes of total fish revealed the summary of relative risk (RR) of 0.97 (95% CI: 0.86–1.10) for total, 1.01 (95% CI: 0.91–1.13) for advanced, and 0.90 (95% CI: 0.72–1.12) for localized prostate cancer, indicating no significant association. Moreover, in summary RR was 0.55 (95% CI: 0.33–0.92) for prostate cancer mortality and 0.84 (95% CI: 0.65–1.10) for prostate cancer progression, indicating an inverse association between fish intake and prostate cancer mortality. In addition, in the dose–response analyses, each 20 g/day increase in total fish intake was associated with a 12% lower risk of prostate cancer mortality. They conclude the protective association between total fish intake and the risk of prostate cancer mortality (Eshaghian et al.).

Furthermore, the clinical significance of nutraceuticals in neurodegenerative diseases and cancer has drastically increased. Thus, nutraceuticals could play a very important role as prophylactic treatment for neurological interventions and cancer. On the other hand, role of nutraceuticals in cancer cells inhibition is well recognized, and at least experimentally in the initial stages of carcinogenesis. However, in fact this area of study still requires further in-depth researches. Therefore, considering the therapeutic advantages of nutraceuticals for the management of cancer and neurodegenerative diseases. Finally, the Guest Editors would like to sincerely thank all the authors and reviewers for their valuable contributions.

## Author contributions

SA: Conceptualization, Writing—original draft, Writing—review & editing. MP: Conceptualization, Visualization, Writing—review & editing. MA: Conceptualization, Visualization, Writing—review & editing.

